# Addressing chronic diseases: a comparative study of policies towards type-2 diabetes and hypertension in selected European countries

**DOI:** 10.1093/eurpub/ckae070

**Published:** 2024-04-04

**Authors:** Chiara Seghieri, Francesca Ferré, Costanza Tortù, Gaia Bertarelli, Christina Mavrogianni, Natalya Usheva, Florian Toti, Luis Moreno, Eirini Agapidaki, Yannis Manios

**Affiliations:** Department L’EMbeDS, Institute of Management, Sant’Anna School for Advanced Studies, Pisa, Italy; Department of Biomedical Sciences for Health, University of Milan, Milan, Italy; Department L’EMbeDS, Institute of Management, Sant’Anna School for Advanced Studies, Pisa, Italy; Department of Economics, Ca’ Foscari University of Venice, Venice, Italy; Department of Nutrition and Dietetics, School of Health Science and Education, Harokopio University, Athens, Greece; Department of Social Medicine and Health Care Organization, Medical University of Varna, Varna, Bulgaria; Department of Internal Medicine, University of Medicine of Tirana, Tirana, Albania; GENUD (Growth, Exercise, Nutrition and Development) Research Group, Universidad de Zaragoza, Instituto Agroalimentario de Aragón (IA2), Instituto de Investigación Sanitaria de Aragón (IIS Aragón), Zaragoza, Spain; Centro de Investigación Biomédica en Red de Fisiopatolo gía de la Oesidad y Nutrición (CIBEROBN), Instituto de salud Carlos III, Madrid, Spain; Ministry of Health, Athens, Greece; Department of Nutrition and Dietetics, School of Health Science and Education, Harokopio University, Athens, Greece; Institute of Agri-food and Life Sciences, Hellenic Mediterranean University Research Centre, Heraklion, Greece

## Abstract

**Background:**

Type-2 diabetes (T2D) and hypertension (HTN) are two of the most prevalent non-communicable diseases (NCDs): they both cause a relevant number of premature deaths worldwide and heavily impact the national health systems. This study illustrates the impact of HTN and T2D in four European countries (Albania, Bulgaria, Greece and Spain) and compares their policies towards the monitoring and management of HTN and T2D and the prevention of NCDs as a whole. This analysis is conducted throughout the DigiCare4You Project (H2020)—which implements an innovative solution involving digital tools for the prevention and management of T2D and HTN.

**Methods:**

The analysis is implemented through desk research, and it is enriched with additional information directly provided by the local coordinators in the four countries, by filling specific semi-structured forms.

**Results:**

The countries exhibit significant differences in the prevalence of HTN and T2D and available policies and programs targeted to these two chronic conditions. Each country has implemented strategies for HTN and T2D, including prevention initiatives, therapeutic guidelines, educational programs and children's growth monitoring programs. However, patient education on proper disease management needs improvement in all countries, registries about patients affected by HTN and T2D are not always available, and not all countries promoted acts to contain the increasing rates of risk factors related to NCDs.

**Conclusions:**

While political awareness of the risks associated with HTN, T2D and NCDs in general is growing, there is a collective need for countries to strengthen their policies for preventing and managing these chronic diseases.

## Introduction

The World Health Organization (WHO) states that non-communicable diseases (NCDs)^1^^,^^2^ are enduring conditions not transmissible between individuals, arising from a combination of genetic, behavioral and environmental factors. Some risk factors for NCDs are unmodifiable, while others are modifiable, such as unhealthy nutrition, physical inactivity, direct or indirect exposure to tobacco smoke and excessive alcohol consumption. NCDs contribute significantly to premature deaths globally, also diminishing the overall quality of life.^3^^,^^4^ Furthermore, NCDs exert a substantial impact on national health systems, demanding efficient and enduring healthcare support for affected patients.^5^ As life expectancy rises, the prevalence of NCDs is expected to intensify, especially among older individuals who are likely to suffer from multiple chronic diseases simultaneously.^6^^,^^7^ Consequently, NCDs pose a challenging threat to the stability of national economic systems worldwide.^8^ Among NCDs, type-2 diabetes (T2D) and hypertension (HTN) are two of the most prevalent diseases. Therefore, it is important for all national and sub-national authorities to introduce policies for the prevention and screening of NCDs comprehensively, and particularly for the management of HTN and T2D, which are particularly diffused within the population.^9^ Policies should also consider the novel technologies developed in recent years.^10^

Within this context, it has been approved by the European Commission, an innovative research project named *DigiCare4You*, that intends to assess the effect of the introduction of a novel mobile app on the prevention and management of T2D and HTN. The Digicare4You project involves four countries, two high-income countries (Spain and Greece) and two low-middle wage countries (Bulgaria and Albania).

In this article, we review and discuss all the existing policies for prevention, screening and management of NCDs, with a particular focus on HTN and T2D, implemented by the four countries involved In the DigiCare4You Program, and by their sub-national entities. Findings might support the definition of a set of priorities for prevention and management of these two chronic diseases in each of the four countries.

## Methods

The information presented in this work is collected by examining all the English-written resources, which provide knowledge on the past, present, or planned healthcare policies implemented in the four inspected countries, either at the national level or at the local level. The literature review is further enriched by integrating additional information from the local partners leading DigiCare4You Program in the four implementation countries. The partners collected all information on policies that are available in sources written in local languages and summarized their content in a specific form. The filled forms returned by the local coordinators are all included in the Supplementary data.

## Results

This section provides evidence of the impact of HTN and T2D in Albania, Bulgaria, Greece and Spain and describes all the national and sub-national policies implemented by the four counties for the screening and management of HTN and T2D, as well as for the prevention of NCDs, to face this burden. We start by providing a preliminary overview of the characteristics of the four national healthcare systems. Then, we present data on the impact of HTN and T2D and we outline future goals set by local political authorities concerning the prevention of these two diseases. Subsequently, we delve into the details of policies and programs implemented in each of the four countries.

### The characteristics of the national healthcare systems

Understanding the specific context in which the four healthcare systems have been developed and understanding their characteristics is crucial for exploring their different approaches in addressing NCDs, and—specifically—T2D and HTN.

The Albanian healthcare system is constructed on the Soviet ‘Semashko’ model, characterized by state ownership of public healthcare institutions.^11^^,^^12^ Since 1995, citizens have accessed the healthcare system through the *Mandatory Healthcare Insurance Fund.* Although health insurance is mandatory for all citizens, inequalities persist in the access to healthcare services. The *Primary Health Care* system is organized through a network of health-service providers present in all Albanian municipalities, offering basic health services. Politicians recognize the need for substantial reform in the healthcare system, as it is still considered one of the most unequal in Europe^12^ and is not universal: only children, retirees and the disabled have free healthcare. Employed individuals must pay a significant premium to obtain a health insurance card.^13^

Bulgaria underwent a profound transformation from a centrally planned economy to a free-market economy in the 1990s, and this transition also impacted the healthcare system.^14^ The Bulgarian healthcare system is highly centralized. Politicians proposed several reforms aimed at decentralizing the healthcare system, reducing costs and monitoring public spending, even if initiative in resuming reforms was partially halted due to political instability.^14^^,^^15^ However, over two main waves of reform since the end of communism, significant changes occurred. The first wave (1989–96) abolished the State monopoly, introducing the concept of an insurance system. The implementation of *Social Health Insurance* (SHI) happened in the second wave (1997–2002), establishing the *National Health Insurance Fund* (NHIF) with contractual relations to healthcare providers. Although the system operates under an insurance model with compulsory SHI and a Voluntary Health Insurance, notable gaps persist in population coverage. Primary care is mainly managed by general practitioners, but the private sector also plays a significant role in healthcare delivery, contributing to a high rate of hospital admissions in the country.^16^ The national political authorities set the health policy agenda through the *National Health Strategy*, while *Regional Health Inspectorates* (RHI) are responsible for implementing state health policies at the regional level.

The Greek healthcare system comprises a *National Health System* (ESY), a *Compulsory Social Insurance* (SHI) and a private healthcare system.^17^^,^^18^ The *National Organization for the Provision of Health Services* (EOPYY), established in 2011, monitors healthcare services acquisition, while the Ministry of Health is responsible for regulating both ESY and EOPYY. Despite funding cuts due to the 2011 economic crisis, Greece achieved comprehensive health insurance coverage. A 2016 reform introduced Primary Health Care units (*TOMYs*), enhancing primary care services, especially for underinsured vulnerable groups. Challenges persist in primary care, including issues of access, continuity and coordination. Primary pediatric care involves collaboration between general practitioners and pediatricians, with specialized centers offering comprehensive pediatric and maternal healthcare services.

In Spain, the *National Health System* (NHS), ensures universal coverage through a decentralized administration ruled by *17 Autonomous Communities* (ACs).^19^^,^^20^ The system comprises three subsystems: the universal NHS, the mutual funds for civil servants and related groups, and the mutualities targeted to accidents and occupational diseases. The NHS emphasizes universality and equality, with the national government coordinating overall efforts and ACs managing regional planning. Primary care is delivered by public providers, including specialized family doctors and nurses, fostering continuity of care. Spain extends free healthcare services, encompassing pediatric care, vaccinations and certain specialized visits, to all resident children, with additional support for those with physical or mental disabilities.

Table 1 summarizes the main characteristics of the health systems in the four countries. The features that have been considered to characterize the systems are (i) the main source of financing, (ii) health spending source (public or private) and (iii) the governance of the health systems. Data presented in table 1 are available in the reports describing the characteristics of the healthcare systems in the four countries.^11–20^

**Table 1:** National health systems—comparison

	Albania	Bulgaria	Greece	Spain
Main source of financing	Social health insurance	Compulsory social health insurance and voluntary health insurance	Tax-based and social health insurance	Tax-based national health system
Health spending source: public/private	Mainly public	Mixed: high share of out of pocket (38%)	Mixed: high share of out of pocket (35%)	Mainly public
Governance	Centralized	Centralized	Mainly centralized	Decentralized

### HTN and T2D: current landscape and future goals

Table 2 reports some data on the diffusion of HTN and T2D and on the overall burden of NCDs, in the four countries. Specifically, it compares the (i) the prevalence of diabetes and HTN, (ii) the percentage of deaths caused by NCDs and (iii) the percentage of chronic patients. Table 2 suggests that both T2D and HTN are widely diffused in the four countries: T2D particularly affects people living in Albania and Spain, while Bulgarian citizens particularly suffer from HTN.

**Table 2:** NCDs Albania, Bulgaria, Greece and Spain

	Albania	Bulgaria	Greece	Spain
Type-2 diabetes^a^ (T2D)	10.2	7.4	6.4	10.3
Hypertension^b^ (HTN)	27.7^c^	28.4	19.1	19.2
Cause of death, by NCDs^d^	94	95	83	91
Chronic patients^e^	23.22^f^	21	24	31

a Percentage of people ages 20–79 with diabetes in 2021, provided by the World Bank Data Catalogue and collected by Atlas.^21^

b Age-standardized prevalence of HTN in 2015.^22^

c Data on the prevalence of HTN are not available for Albania. The estimated prevalence of hypertensive patients is obtained by evaluating the results presented by Gjonça et al.^23^

d Percentage of deaths due to NCDs in 2019, provided by the World Bank Data Catalogue.^24^

e Percentage of adults with a self-reported longstanding illness in 2019.^25^

f Data on the % of self-reported chronic patients in 2016 are not available for Albania in statista.com. The estimated prevalence of chronic patients is obtained by evaluating the results presented by Kraja et al.^26^

**Figure 1:**
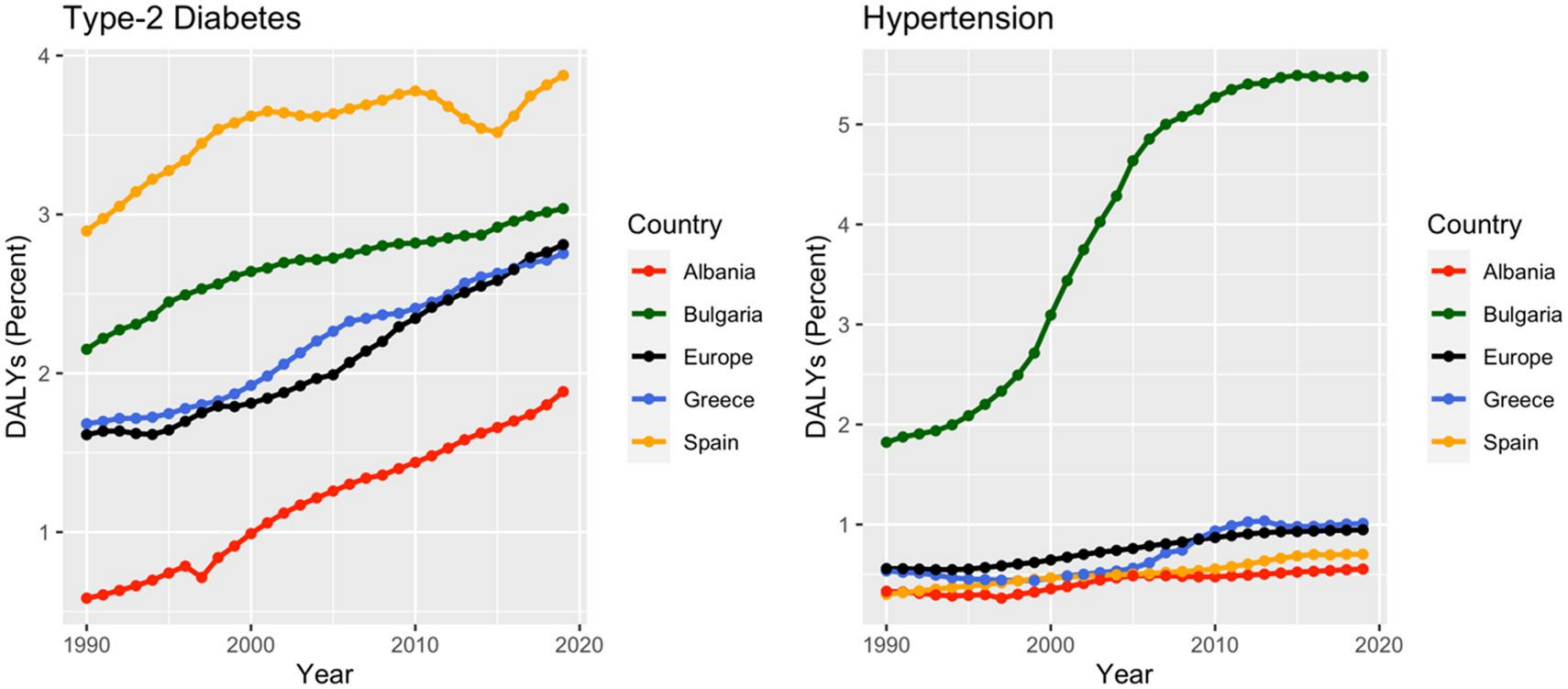
DALYs’ trends for HTN and T2D in Albania, Bulgaria, Greece and Spain, 1990–2019. Source: Institute for Health Metrics and Evaluation

All four countries face challenges in halting the rising rates of T2D and HTN, along with addressing risky behaviors contributing to NCDs. In Albania, the percentage of 11- and 15-year-olds meeting WHO-recommended daily physical activity is low, and diabetes prevalence has doubled in the past decade, surpassing the EU average.^16^^,^^27^^,^^28^ Bulgaria, with high rates of smoking, alcohol consumption and poor diet, records the lowest life expectancy in the EU, with increased prevalence of both T2D and HTN.^29^ Greece struggles with high rates of smoking and obesity,^30^ and Spain has a substantial population affected by chronic diseases, especially T2D.^31^ To assess the global burden of these two chronic conditions, we examine disability-adjusted life years (DALYs) due to T2D and HTN, using data available in *the Global Burden of Diseases* (*GBD*) dataset,^32^ published by the *Institute for Health Metrics and Evaluation* (figure 1). The analysis reveals a significant increase in DALYs attributed to HTN and T2D from 1990 to 2019 in all four countries, with HTN notably impacting the Bulgarian population.

All four countries, recognizing the significant impact of HTN and T2D have set specific quantitative targets for prevention. These goals either directly address the reduction of HTN and T2D prevalence or are tailored to mitigate the risk factors associated with NCDs. In Bulgaria, the *National Programme for the Prevention of Chronic Non-Communicable Diseases 2021–2025* outlines comprehensive targets addressing both risk factors and disease prevalence. The program targets a 5% drop in smoking and a 10% cut in low physical activity. It also aims for a 10% relative decrease in T2D and a 5% reduction in HTN within the 25–64 age group. Greece's *National Action Plan* aims to decrease childhood obesity and smoking rates. Spain's *Health Promotion and Prevention Strategy* targets a 2-year increase in life expectancy in good health by 2025. In Albania, a specific program targeted to NCDs aims to achieve a 10% reduction in premature mortality by 2025 and a 10% reduction in adult HTN prevalence, while also striving to halt the rise of diabetes.

### Policies towards HTN and T2D

To reach these targets, the national authorities in the four countries proposed policies and programs to improve the management of patients affected by HTN and T2D and to prevent the diffusion of NCDs as whole. These policies broadly include (i) the institution of children monitoring programs to detect early signs of chronic conditions, (ii) the promotion of guidelines to more effectively treat patients affected by HTN and T2D and (iii) the dissemination of action plans to reduce the prevalence of HTN and T2D, and to decrease the diffusion of risk factors related to NCDs. Details about the policies are included in specific forms that were accurately filled by the local stakeholders and that are available in the Supplementary data.

In Albania, the Child Primary Care System, which is highly integrated with Family Medicine Service, is responsible for the prevention and the management of the most common diseases for children aged between 0 and 14 years.^33^ In urban areas, the primary care for children is also delivered by specific consulting centers, which provide services related to child nutrition and growth monitoring. There exist a specific children growth monitoring program, targeted to children aged between 6 and 15 years old: it assesses the child’s medical history together with the corresponding record of completed vaccinations and aims to detect early signs of chronic conditions. Given the increasing rates of HTN and T2D and of NCDs, political authorities push for a stronger awareness on healthy lifestyles and the Government studied some specific interventions against HTN and T2D. In 2018, the Albanian Diabetes Association disseminated the *Guidelines for the screening, diagnosis, prevention, and treatment of Diabetes and its complications*, to help the healthcare professionals in treating diabetic patients according to their best personalized scheme. In 2019, the University of Medicine Tirana and University Hospital center ‘Mother Theresa’ further expanded these guidelines and promoted a protocol for managing patients with T2D. Recently, the *National Plan for Control and Prevention of NCDs* instituted a National Committee entirely dedicated to NCDs and designated NCD local points, who plan activates in coordination with the NCD unit at the Institute of Public Health. The program—also known as ‘Si Je?’ *(How are you?)*—was launched in 2015 and intended to shift the focus from reactive care to prevention. It allows all Albanians aged 35–70 years to receive a free, yearly basic health examination at their local health center,^18^ together with specialized medical consultations. There is also an existing action plan—*the Food and Nutrition Policy Discussion Paper 2013–2020*—which promotes the importance of healthy nutrition.

In Bulgaria, there is a *National Program for Prevention of Non-Communicable Diseases 2021–2025* which promotes population’s health and monitors indicators about biological and lifestyle risk factors for chronic diseases. However, there are not accurate statistics that monitor the spread of NCDs, and, particularly, of T2D and HTN, and there are no existing guidelines for the management of these two chronic conditions.^29^^,^^34^ A registry of patients affected by T2D in the Republic of Bulgaria existed only for the period 2013–19, when it was maintained by the University Specialized Hospital for Active Treatment of Endocrinology. The national authorities are particularly committed in encouraging the development of educational programs for healthy nutrition for children: the information is communicated through proper educational modules that are shaped based on the age of the children they are targeted to. There is a program for children growth monitoring targeted to scholars aged 7–18: aggregated results are analysed and shared with the RHI. In 2019, the Bulgarian Ministry of Health established a National agreement between the NHIF and the providers of medical care: according to this agreement, people are periodically monitored through medical check-ups to detect early signs of chronic conditions. The three main agents that play a role in prevention against NCDs are the general practitioners, who monitor, refer for tests and educate patients on risk factors and treatment of diseases, the National Centre for Public Health and Analyses and the regional health inspectorates who both conduct disease prevention campaigns to improve health literacy and promote healthy lifestyles.

In Greece, the National Government is particularly committed to prevent children from chronic diseases. Therefore, it introduced in 2014 a form of children development monitoring, called *Individual student health record* (*ISHR*). To have their children registered at school, parents need to provide a brief medical examination document completed by pediatricians. This document provides information on the child’s medical history, together with their record of vaccinations. Patients affected by T2D are regularly assisted in specialized departments instituted in major hospitals as well as in outpatient clinics. However, the work conducted by Liatis et al.^35^ provides an overview on the current T2D management, and states that a significant quote of diabetic patients achieves low glycemic control and that there is ample room for optimizing the timing of add-on therapies, so to minimize the overall risk of complications. A complete national plan for the prevention and management of T2D was established in 2012. The document, named *National Action Plan for the Prevention and Treatment of Diabetes Mellitus* and its complications contains suggestions about how to adopt a healthy lifestyle, with the global objective of informing the public on risks of T2D. In 2021, the Hellenic Diabetes Association disseminated *Guidelines for the management of patients with Diabetes Mellitus*, to guide the healthcare professionals in treating T2D according to personalized approach, derived through an appropriate algorithm, which takes as input the whole patient’s profile. In addition, the document also provides dietary and physical activity guidelines together with indications on how to deal with possible comorbidities. Similarly, Hellenic authorities and scientific associations have strived to improve the prevention of HTN, while also proposing guidelines to treat this disease. In particular, the Hellenic Society of Hypertension translated the international guidelines for the management of HTN, which were previously disseminated by the European Society of Cardiology & European Society of Hypertension. Moreover, the Working Group for Cardiovascular Diseases summarized the most effective diagnostic and therapeutic protocols to treat HTN and provided data-driven suggestions for a more effective treatment of chronic cardiovascular diseases as a whole.

In Spain, the Ministry of Health has an appropriate area dedicated to health promotion and disease prevention. This area works in close collaboration with the ACs and other organizations to implement prevention and control policies. Recent reforms in the healthcare sector developed guidance to reduce the diffusion of risk factors related to NCDs. For instance, the NAOS Strategy, launched in 2005, aims to reduce the growth of obesity. This strategy was further strengthened by the law on food security and nutrition adopted in 2011. In 2019, the Ministry of Finance has also implemented two reforms to increase taxes of alcohol and tobacco. Spain particularly suffers from the increasing burden of NCDs on the healthcare system.^31^^,^^36^ In 2012, the national Government supported a novel program named *Diabetes Strategy of the National System of health* which intended to improve the screening and prevention of T2D and to enhance the quality of life of patients. A proper national diabetes plan is in place since 2007 and provides general guidelines to stimulate the implementation of regional programs for prevention, early diagnosis and efficient treatment of T2D. The role of ACs is indeed important in the management of T2D and HTN, and of NCDs as a whole: most of them have advisory councils for T2D, plan education programs for T2D and promote initiatives to prevent NCDs. Some ACs directly implemented policies against T2D. As example, in 2018, the Government of the Autonomous Region of Aragon introduced a nurse care plan for diabetic patients: the project collected information on the lifestyles and health status of diabetic and developed a health plan to advise standardized nursing actions for specific segments of patients. A few years later, in 2021, the local authority of the Region of Aragon supported a proper program, named *Comprehensive Diabetes Mellitus Care Program*, to support the organization of preventive initiatives against T2D and to promote the collaboration within health workers in the development of a more effective strategy for detecting and monitoring diabetic patients.

Table 3 summarizes the main findings described in this paragraph and provides an illustrative comparison of the main policy actions tackling NCDs in the four countries. In particular, it reports the existing policies about the (i) prevention of NCDs, (ii) screening and (iii) management of T2D and HTN, implemented by the four countries.

**Table 3:** Overview of the policy actions tackling NCDs in Albania, Bulgaria, Greece and Spain—illustrative comparison of the results

Policy actions against NCDs	Albania	Bulgaria	Greece	Spain
Prevention	Guidelines ADA, children growth monitoring program	National Program for Prevention of NCDs GPs program for preventive check-ups	Individual student health record	Strategy for Nutrition, Physical Activity and Prevention of Obesity (NAOS)
Screening	Guidelines ADA, ‘Si Je?’	Programs for screening Agreement between the NHIF and the providers of medical care	National plan for T2D and HTN	Diabetes Strategy (Ministry of Health)
Management	Guidelines ADA	Agreement between the NHIF and the providers of medical care	National plan for T2D and HTN, Guidelines for the management of T2D and HTN	Diabetes Strategy (Ministry of Health), National Diabetes Plan

## Discussion

All the four countries face an increasing burden of both HTN and T2D, so political authorities are encouraged to improve their healthcare policies targeted to the screening and management of these two diseases, and to the prevention of NCDs as a whole. The four countries show relevant discrepancies with respect of the organization of the national healthcare system, and these differences also impact their strategy to address HTN and T2D: Bulgaria and Albania have a highly centralized system that still presents significant inequalities in access to care and gaps in the continuity of care; Greece is characterized by a mixed healthcare system, and Spain has a public decentralized healthcare system, where the National Government and the local regions co-operate in addressing HTN and T2D.

The national healthcare systems of the four countries all face the challenge of managing patients affected by HTN and T2D, while also attempting to improve prevention of NCDs as a whole: to this aim, the four countries implement policy strategies, which translate into different laws. We highlight some positive elements that should be strengthened. First, all the four countries have a program for the monitoring of child growth: this enables health workers to detect early risk factors, which may cause the onset of a chronic disease and, therefore, plays a pivotal role in improving screening activities, as also highlighted by De Onis et al.^37^ Second, all countries provide common guidelines for the prevention, screening and management of T2D and HTN to support all the health workers in following medical recommendations that are validated by the scientific community. This is highly important to reduce the burden due to HTN, T2D and NCDs as a whole, as pointed out by Kovacs et al.^38^ Third, similarly to what obtained by Heller et al.,^39^ the political awareness of the risks related to NCDs as a whole is increasing, as proved by the increasing number of policies towards NCDs approved in recent years.

However, there are also some critical elements that emerged from this comparative analysis. First, national registries for patients affected by HTN and T2D are not available in all the examined countries, and this prevents authorities from accurately mapping the diffusion of these two chronic diseases. Second, not in all countries citizens can easily access to specialized visits and there is a lack of trained healthcare professionals that accurately monitor patients affected by HTN and T2D in the everyday management of their disease. Third, there is no specific budget allocated specifically to the screening and the management of HTN and T2D, and to the prevention of NCDs in general: the resources for healthcare systems, already impacted by the COVID-19 pandemic, are primarily directed towards ensuring the provision of the essential level of care. Finally, not all countries have effectively promoted strategies to contain the increasing rates of risk factors related to NCDs, such as smoking and obesity^40^: in addition to the informative campaigns, governments should implement specific policies to discourage people from smoking and eating unhealthy food.

To sum up, this analysis points out that the in the European countries we included in the analysis the political awareness of the risks related to HTN and T2D is raising: all the four countries have recently promoted policies to improve the management of HTN and T2D, and to prevent NCDs as a whole. However, additional efforts are needed to update all the existing policies and making them more effective, and to improve the quality and the general organization of the national healthcare systems.

## Supplementary Material

ckae070_Supplementary_Data

## Data Availability

The data underlying this article are available in the article and in its online supplementary material. Keypoints The analysis compares policies for hypertension (HTN) and type-2 diabetes (T2D) in four European countries. Both healthcare systems and policies for HTN and T2D present differences in the countries. The political awareness of the risks related to HTN and T2D is increasing. Preventive policies should be implemented to reduce the burden of HTN and T2D. The diffusion of HTN and T2D should be more precisely monitored.
