# Supplementary material for: Addressing chronic diseases: a comparative study of policies towards type-2 diabetes and hypertension in selected European countries
Source: Eur J Public Health. 2024 Apr 4;34(4):781–6. doi: 10.1093/eurpub/ckae070 (PMC11299210; doi:10.1093/eurpub/ckae070)
Supplement: ckae070_Supplementary_Data [file ckae070_supplementary_data.docx]

Supplementary materials for “Addressing chronic diseases: a comparative study of policies towards Type-2 Diabetes and Hypertension in selected European countries”

A.1 Forms for Albania

**Questionnaire for each policy / programme document separately**

1. On which country do you report? **Albania**

2. What are the main policy / programme documents in which the vision and major plans for screening, prevention, and management of T2D and HTN in your country have been formulated and T2D or HTN have been mentioned explicitly?

Please mention name and publication year of the policy document and, for each document, the main points concerning screening, prevention, and management of T2D and HTN. You should also include policies on children’s growth/health assessment.

*please fill in the form separately for T2D, HTN and Children’s growth/health monitoring.*

See next table for each policy document / programme

| **Name of the policy / programme document (year / url / publication)** | **Name: National Plan for Control and Prevention of non – communicable diseases**  [Programi Kombëtar për Parandalimin dhe Kontrollin e Sëmundjeve Joinfektive në Shqipëri 2016-2020](http://www.ishp.gov.al/wp-content/uploads/2017/05/programi-kombetar-SJI-.pdf)  **Year: 2016**  **URL:** <http://www.ishp.gov.al/dokumenta-strategjike/>  **Publication:**  Institute of Public Health |
| --- | --- |
| **Focus on** | X T2D  X HTN  Children’s growth/ health monitoring |
| **This policy/programme is relevant to:** | X the 1^st^ stage screening  X the 2^nd^ stage screening  X the intervention |
| **In action / Planned** | X In action  Planned |
| **In action / Planned date** | 2016 |
| **Initiator / Stakeholder** | X government  local authority  NGO  community  other, specify:    Detailed Information: Under the name: “Si je?” (How are you? in English) this document is part of a national campaign, whose purpose is to inform and educate the public about diabetes. It is created by the Ministry of Health, the Public Health Institute, and the National Health Fund, in collaboration with World Health Organization (WHO). |
| **Level of policy intervention / implementation** | X national  regional  local  community  school    Detailed Information: All adults 35-70 years old, living in Albania, is included in this program, with the idea of an annual check-up, consultations with Primary Health care, and if necessary, consultations with specialists (mostly endocrinologist, and cardiologist). It is a national action plan that contains recommendations and policy initiatives but due to various reasons, including political, it is not fully implemented yet. And the situation with COVID-19, has stopped for more than a year its implementation. |
| **Category** | legislation  X protocol  X guideline  subsidy  X programme  Can’t tell |
| **T2D and HTN focus (more than one answer possible)** | General  Screening  Prevention  Management  Screening + Prevention  Screening + Management  X Screening + Prevention + Management  Prevention + Management |
| **Policy / intervention targets / policy goals / determinants addressed** **(more than one answer possible)** | X Improving screening of T2D or HTN  X Improving prevention of T2D or HTN  X Improving management of T2D or HTN  Monitoring growth and development of children in schools or communities  Empowerment  Improving (health) services  Influencing policy  Promoting (please specify below)  Reducing (please specify below)    Detailed information: Diabetes is a disease that concerns the whole society and requires collective approach. The central goal of the document is to raise awareness not only to the patient and the health care professionals (HCPs), but also to the general public.  To prevent type 2 diabetes, goals for prevention and management are set. More specifically goals for body weight, physical activity, nutrition, smoking, enhancement of prevention interventions as well as raising awareness for patients and HCPs, providing specialized medical treatment, and upgrading of facilities and equipment. |
| **Measurement / evaluation / monitoring tools** | The implementation of the goals is evaluated and monitored by the Ministry of Health, and an expert’s panel. |
| **Target group (i.e. general population, children <18,…)** | General population 35-70 years old. |
| **Description (description of the policy including the health profession disciplines occupied in the policy / program)** | The content of the document is:  - definition of diabetes  - risk factors for DM  - epidemiological data  - policy planning for prevention and treatment  - financial burden of DM |
| **Implementation details**  **(more than one answer possible)** | Phases:  problem definition  agenda setting  policy development  X implementation  X policy evaluation    Detailed information: The implementation process discussed in the document includes target setting for monitoring the effectiveness of the plan, but not specific steps on how to achieve these targets. The bodies/organization/institutes involved in programmes for the prevention and management of diabetes will have to report their actions and progress at the Ministry of Health in a regular basis (every 6 months). |
| **Lessons learned** | Not fully implemented, therefore this section cannot be answered at the moment. |
| **Are you aware of data sources aimed at monitoring variables relevant to this policy / programme?**  **If yes, which ones?** | This action plan is not fully implemented. We have received only fragmentary data about the number of persons screened every year, but not any data regarding the importance of cost- efficiency of early diagnosis or prevention. |

| **Name of the policy / programme document (year / url / publication)** | **Name: Guideline for the screening, diagnosis, prevention, and treatment of Diabetes and its complications**    Year: 2018  Publication: Albanian Diabetes Association |
| --- | --- |
| **Focus on** | X T2D  X HTN  Children’s growth/ health monitoring |
| **This policy / programme is relevant to:** | X the 1^st^ stage screening  the 2^nd^ stage screening  X the intervention |
| **In action / Planned** | X In action  Planned |
| **In action / Planned date** | 2019 |
| **Initiator / Stakeholder** | government  local authority  NGO  community  X other, specify: Scientific Association    Detailed Information: **Albanian Diabetes Association** |
| **Level of policy intervention / implementation** | X national  regional  local  community  school    Detailed Information: Guideline with the aim to help health care professionals (HCPs) to achieve the best possible result/to treat diabetic patients. |
| **Category** | legislation  protocol  X guideline  subsidy  programme  Can’t tell |
| **T2D and HTN focus (more than one answer possible)** | General  Screening  Prevention  Management  Screening + Prevention  Screening + Management  X Screening + Prevention + Management  Prevention + Management |
| **Policy / intervention targets / policy goals / determinants addressed**  **(more than one answer possible)** | X Improving screening of T2D or HTN  X Improving prevention of T2D or HTN  X Improving management of T2D or HTN  Monitoring growth and development of children in schools or communities  Empowerment  Improving (health) services  Influencing policy  Promoting (please specify below)  Reducing (please specify below)    Detailed information: This document is created by the Scientific Committee of Albanian Diabetes Association, and provides guidelines for the screening, diagnosis, the treatment, monitoring, and follow-up of diabetes and accompanying comorbidities. The target of this document is the guidance of HCPs in order to apply the best personalized scheme and achieve the best treatment for the patient, based on scientific clinical evidence (“Evidence Based Medicine”). Dietary, physical activity, glycemic and pharmaceutical targets are specified according to the patient’s profile. It includes guidelines not only for type 2 diabetes, but for type 1 and Gestational Diabetes too.    Regarding Type 2 Diabetes prevention, it is more focused in people with high risk for T2D (ex. obese or pre-diabetic patients), since no specific actions are adopted up to date at a national level. The effectiveness of the intervention for high-risk patients is well documented, especially for people with impaired glucose tolerance (IGT). High risk individuals are identified after diagnostic test (fasting glucose and sugar curve) for diabetes. Dietary guidelines are an indispensable part of T2D prevention and treatment. |
| **Measurement / evaluation / monitoring tools** | In Albania, till now, there is no official guideline regarding diabetes management. The present document “Guidelines for the management of patients with Diabetes Mellitus” is the main guide for each health professional during their daily clinical practice for the management of patients with diabetes.    The evaluation and the monitoring of the diabetic patient are performed on a personalized basis. The HCPs, according to the medical history of the patient, the progress, the response to the treatment, generally his/her profile, can prescribe diagnostic tests, treatments, and schedule further consultations, based on this guidelines. |
| **Target group (i.e. general population, children <18,…)** | General population |
| **Description (description of the policy including the health profession disciplines occupied in the policy/program)** | The document is referred to all people with diabetes, with pre-diabetes, T2D, or T1D.  Content: definition, classification, and treatment of diabetes, prevention, dietary and physical activity guidelines, treatment procedures either in a hospital or at home, treatment of diabetes and its comorbidities and implications. |
| **Implementation details**  **(more than one answer possible)** | Phases:  X problem definition  agenda setting  X policy development  X implementation  policy evaluation    Detailed information: these guidelines provide algorithms for diagnosis, treatment, and monitoring of patient’s progress. |
| **Lessons learned** | As it is not yet made official by the Ministry of Health and National Health Fund, HCP have difficulties to implement it in the daily practice, due to some discrepancies with the actual protocol for type 2 Diabetes by Health Fund. |
| **Are you aware of data sources aimed at monitoring variables relevant to this policy / programme?**  **If yes, which ones?** | No, there is no legislation or monitoring of these guidelines on a national level. |

| **Name of the policy / programme document (year / url / publication)** | **Name: Protocol for managing patients with type 2 Diabetes Mellitus**    Year: 2019  URL: <https://fsdksh.gov.al/project/diabeti/>  Publication: National Health Fund |
| --- | --- |
| **Focus on** | X T2D  HTN  Children’s growth/ health monitoring |
| This policy/programme is relevant to: | the 1^st^ stage screening  the 2^nd^ stage screening  X the intervention |
| **In action / Planned** | X In action  Planned |
| **In action / Planned date** | 2019 |
| **Initiator / Stakeholder** | X government  local authority  NGO  community  other, specify:    Detailed Information:  A concise protocol guiding health care professionals (HCPs) to achieve the best possible result/to treat T2D patients. |
| **Level of policy intervention/ implementation** | X national  regional  local  community  school    Detailed Information: This document is the product of the specialists at the University of Medicine Tirana, and University Hsopital center “Mother Theresa”, and provides guidelines for the diagnosis, treatment, monitoring, and follow-up of diabetes. The target of this document is the guidance of HCPs in order to apply the best personalized scheme and achieve the best treatment for the patient. Dietary, physical activity, glycemic and pharmaceutical targets are specified according to the patient’s profile. |
| **Category** | legislation  X protocol  X guideline  subsidy  programme  Can’t tell |
| **T2D and HTN focus (more than one answer possible)** | General  Screening  Prevention  X Management  Screening + Prevention  Screening + Management  Screening + Prevention + Management  Prevention + Management |
| **Policy / intervention targets / policy goals / determinants addressed**  **(more than one answer possible)** | Improving screening of T2D or HTN  Improving prevention of T2D or HTN  X Improving management of T2D or HTN  Monitoring growth and development of children in schools or communities  Empowerment  Improving (health) services  Influencing policy  Promoting (please specify below)  Reducing (please specify below)    Detailed information:  This protocol for a better management of Type 2 diabetes, is a wider and more detailed version of the previous one. Its aim is to give some guidelines about the initiation, follow-up, and intensification therapy for T2DM, regarding only the metabolic control, without any emphasize for other cardiovascular risk factors. |
| **Measurement / evaluation / monitoring tools** | The implementation of the goals is evaluated and monitored by the National Health Fund and Ministry of Health. |
| **Target group (i.e. general population, children <18,…)** | General population |
| **Description (description of the policy including the health profession disciplines occupied in the policy/program)** | The content of the document is:  - definition of diabetes  - risk factors for DM  - epidemiological data  - policy planning for prevention and treatment  - financial burden of DM  - implementation process-funding |
| **Implementation details**  **(more than one answer possible)** | Phases:  problem definition  agenda setting  policy development  X implementation  policy evaluation    Detailed information:    The implementation process discussed in the document includes target setting for monitoring the effectiveness of the plan, but not specific steps on how to achieve these targets. The bodies/organization/institutes involved in programmes for the prevention and management of diabetes will have to report their actions and progress at the Ministry of Health. |
| **Lessons learned** | Difficult to change habits of Primary Care Physicians. Only a few of them are using and implementing in daily practice this protocol. |
| **Are you aware of data sources aimed at monitoring variables relevant to this policy / programme?**  **If yes, which ones?** | As I have already mentioned, due also to COVID-19 pandemic, the protocol is not widely use, or any assessment has been caried by the Fund or Ministry of Health |

| **Name of the policy / programme document (year / url / publication)** | **Name:** “Individual pupils health records”      **Year: 2010**  **URL:**    **Publication: Ministry of Health, Ministry of Education** |
| --- | --- |
| **Focus on** | T2D  HTN  X Children’s growth/ health monitoring |
| **This policy/programme is relevant to:** | X the 1^st^ stage screening  the 2^nd^ stage screening  the intervention |
| **In action / Planned** | **X** In action  Planned |
| **In action / Planned date** | 2010 |
| **Initiator / Stakeholder** | X government  local authority /community  NGO  other, specify: Scientific Association    Detailed Information:  Legislation from the government MoH, MoE |
| **Level of policy intervention/ implementation** | X national  regional  local/community  X school    Detailed Information: A useful tool for screening and monitoring of pupil’s health throughout school but with mandatory completion during the registration of children at school at kindergarten, 1^st^ and 5^th^ class of primary school, and 4^th^ class of secondary school. |
| **Category** | X legislation  protocol  guideline  subsidy  programme  Can’t tell |
| **T2D and HTN focus (more than one answer possible)** | Screening  Prevention  Management  X Screening + Prevention  Screening + Management  Screening + Prevention + Management  Prevention + Management |
| **Policy / intervention targets / policy goals / determinants addressed**  **(more than one answer possible)** | Improving screening of T2D or HTN  Improving prevention of T2D or HTN  Improving management of T2D or HTN  X Monitoring growth and development of children in schools or communities  Empowerment  Improving (health) services  Influencing policy  Promoting (please specify below)  Reducing (please specify below)    Detailed information: The recording program serves the prevention and protection of student’s health. It is also a communication channel between the school units and the doctor. |
| **Measurement / evaluation / monitoring tools / health professionals involved (e.g. diabetologists, nurses, general practitioners)** | It assesses the child’s medical history (including and family history) and vaccination records. What is more, it consists of body measurements and, clinical examination of skin, vision, oral cavity, teeth, heart listening (murmurs, tones, rhythm), respiratory system, genitals, nervous and musculoskeletal system, and scoliosis. The medical examination is performed mainly by paediatrician. If the latter is not available, general doctors or physicians can also carry out the examination. |
| **Target group (i.e. general population, children <18)** | Children <18 years old, at kindergarten, at 1^st^ and 5^th^ class of primary school, and 4^th^ class of secondary school. |
| **Description** | The role of this document is the screening for the early detection of diseases, and generally the physical and psychosocial support of the child. Further medical control follows in case of special medical indications. It is a prerequisite for the participation of students in sports and other activities at school. |
| **Implementation details**  **(more than one answer possible)** | Phases:  problem definition  agenda setting  policy development  X implementation  policy evaluation    Detailed information:  It is established as a necessary document for the enrolment in the kindergarten, the 1^st^ and 5^th^ class of the primary school, and 4^th^ class of secondary. It also serves as a medical document for the monitoring of childrens’ health during its presence in primary and secondary education. The document is archived at school’s files and a copy is saved at child’s health booklet. |
| **Lessons learned** | Useful record for monitoring schoolchildren’s growth and development. Not all the regions are using it regularly.  Should be electronically kept (currently it is only filled in a paper document) for ease of reference and to serve as a surveillance system. |
| **Are you aware of data sources aimed at monitoring variables relevant to this policy / programme?**    **If yes, which ones?** | According to the legislation, in case student’s health status changes throughout the years, the document is updated under the responsibility of parents or guardians. Since it is a prerequisite for child’s enrollment, it is guaranteed that it will be filled. However, in practice there is not an official monitoring body to assess if this is performed. |

**A.2 Forms for Bulgaria**

**Questionnaire for each policy / programme document separately**

1. On which country do you report? **Bulgaria**

2. What are the main policy / programme documents in which the vision and major plans for screening, prevention, and management of T2D and HTN in your country have been formulated and T2D or HTN have been mentioned explicitly?

Please mention name and publication year of the policy document and, for each document, the main points concerning screening, prevention, and management of T2D and HTN. You should also include policies on children’s growth/health assessment.

*please fill in the form separately for T2D, HTN and Children’s growth/health monitoring.*

See next table for each policy document / programme

| **Name of the policy / programme document (year / url / publication)** | **Name:** NATIONAL PROGRAMME FOR PREVENTION OF NON-COMMUNICABLE DISEASES  **Year:2021**  **URL:** <https://www.mh.government.bg/media/filer_public/2021/08/09/nacionalna-programa-prevenciq-hnb-2021-2025_htc24ZU.pdf> |
| --- | --- |
| **Focus on** | X T2D  X HTN  Children’s growth/ health monitoring |
| This policy/programme is relevant to: | X the 1^st^ stage screening  X the 2^nd^ stage screening  X the intervention |
| **In action / Planned** | X In action  Planned |
| **In action / Planned date** | 2021-2025 |
| **Initiator / Stakeholder** | X government  local authority /community  NGO  other, specify:    Detailed Information: The program is managed by the National Program Council, which is headed by the Deputy Minister of Health. |
| **Level of policy intervention/ implementation** | X national  regional  local/community  school    Detailed Information: |
| **Category** | legislation  protocol  guideline  subsidy  X programme  Can’t tell |
| **T2D and HTN focus (more than one answer possible)** | Screening  Prevention  Management  Screening + Prevention  Screening + Management  X Screening + Prevention + Management  Prevention + Management |
| **Policy / intervention targets / policy goals / determinants addressed**  **(more than one answer possible)** | X Improving screening of T2D or HTN  X Improving prevention of T2D or HTN  X Improving management of T2D or HTN  Monitoring growth and development of children in schools or communities  Empowerment  Improving (health) services  X Influencing policy  X Promoting (please specify below)  Reducing (please specify below)    Detailed information: In the medium term, the objectives are to maintain levels for indicators (compared to baseline 2020) for morbidity, disability, mortality, frequency of risk factors.  The long-term goals (compared to the baseline for 2020) are to reduce the incidence of hypertension in the age group 25-64 by 5%.  Also, relative reduction in the incidence / prevalence of type 2 diabetes (defined as an elevated blood glucose level ≥ 7.0 mmol / L or individuals with treatment for diabetes) among people over 25 years of age by 10%.  reducing the incidence of acute and chronic complications of type 2 diabetes; hospitalization of type 2 diabetes patients. |
| **Measurement / evaluation / monitoring tools / health professionals involved (e.g. diabetologists, nurses, general practitiones)** | Monitoring and evaluation indicators: age and sex composition of the population; dissemination of lifestyle risk factors (tobacco smoking, alcohol consumption, low physical activity, unhealthy eating); prevalence of biological risk factors (increased arterial pressure, elevated total serum cholesterol, elevated index of body mass (BMI); morbidity / morbidity of the population by reasons, sex and age; mortality of the population by reasons, sex and age; disability of the population by causes, gender and age.  The evaluation of the program is based on the monitoring data. The components for evaluation include significance, adaptability, degree of performance, effectiveness, impact, efficiency (comparison of invested resources- financial, human, etc., with the obtained results).  Health specialists, involved with the program: Bulgarian medicine union, general practitioners, medical specialists, scientific medical societies, physicians/nurses in schools and kindergartens, medical establishments for primary or specialized outpatients and hospital care. |
| **Target group (i.e. general population, children <18,…)** | -Children  -Young people  - Women in reproductive age  - Working age population  - Elderly and old people  - Medical professionals and associated medical/ health professionals  - Non- medical specialists |
| **Description** | The content of the document is:  - Introduction and epidemiological data for NCD (incidence, mortality, burden of diseases)  - frequency of risk factors for NCD  - results of the Programme 2014-2020  - policy planning for prevention and treatment of NCD  - goals of the programme  - responsible institutions and organizations  - indicators for monitoring of the programme |
| **Implementation details**  **(more than one answer possible)** | Phases:  X problem definition  agenda setting  X policy development  X implementation  X policy evaluation  Detailed information: Two national coordinators are responsible for the implementation of the program policy and the achievement of its objectives; manage and participate in the development of the program documents and the work plans, according to the strategies of the program, its goals and tasks; distribute the financial resources provided under the program; coordinate activities in the areas; upon request and on other occasions present the results of the activities of the Ministry of Health; prepare annual information on the program activities. |
| **Lessons learned** | This is a first year of the Programme and data is not available. |
| **Are you aware of data sources aimed at monitoring variables relevant to this policy / programme?**  **If yes, which ones?** | National Center for Public Health and Analysis (NCPHA) <https://ncpha.government.bg/?lang=en>  and National Statistical Institute <https://nsi.bg/en/content/3280/health> |

| **Name of the policy / programme document (year / url / publication)** | **Name: National framework agreement № RD-NS-01-4/23 December 2019 for medical activities between the National Health Insurance Fund and the Bulgarian**  **Medical Union (Suppl. 12);**  Year: 2020-2022  URL:  Publication: |
| --- | --- |
| **Focus on** | X T2D  X HTN  X Children’s growth/ health monitoring |
| **This policy/programme is relevant to:** | X the 1^st^ stage screening  X the 2^nd^ stage screening  the intervention |
| **In action / Planned** | X In action  Planned |
| **In action / Planned date** |  |
| **Initiator / Stakeholder** | X government  local authority /community  NGO  other, specify:    Detailed Information: *Ministry of Health* |
| **Level of policy intervention/ implementation** | X national  regional  local/community  school  Detailed Information: This agreement defines financial, medical, organizational, managerial, informational, and legal-deontological frameworks, in accordance with  which are concluded contracts between the Bulgarian National health insurance fund and the providers of medical care. |
| **Category** | X legislation  protocol  guideline  subsidy  programme  Can’t tell |
| **T2D and HTN focus (more than one answer possible)** | Screening  Prevention  Management  Screening + Prevention  Screening + Management  X Screening + Prevention + Management  Prevention + Management |
| **Policy / intervention targets / policy goals / determinants addressed**  **(more than one answer possible)** | X Improving screening of T2D or HTN  X Improving prevention of T2D or HTN  X Improving management of T2D or HTN  X Monitoring growth and development of children in schools or communities  Empowerment  Improving (health) services  Influencing policy  Promoting (please specify below)  Reducing (please specify below)    Detailed information: The activities of GPs are aimed at the entire population >18 years and specific preventive activities for people with risk factors - hypertension, overweight, obesity, smoking, and others. Prevention of hypertension and type 2 diabetes mellitus is conducted by measuring arterial pressure and testing fasting serum glucose in healthcare providers |
| **Measurement / evaluation / monitoring tools / health professionals involved (e.g. diabetologists, nurses, general practitioners)** | Anthropometrical measurement and assessment of neuropsychological development in children (0-18 years) on regular basis: two times per year for age groups 2-6 years; annually for school children (7-18 years old).  Measurement of SCORE and FINDRISK in patient with risk factors for cardiovascular diseases and type 2 diabetes mellitus.  Health professionals involved - general practitioners, nurses, pediatritians, cardiologists, endocrinologists |
| **Target group (i.e. general population, children <18,…)** | Children ≤18 years – prevention (incl. immunizations) and monitoring of growth and development;  Health insured population > 18 years |
| **Description** | This agreement defines medical, financial, organizational-managerial, informational, and legal-deontological frameworks, in accordance with which are concluded contracts between the NHIF (National Health Insurance Fund) and the providers of medical care. |
| **Implementation details**  **(more than one answer possible)** | Phases:  problem definition  agenda setting  X policy development  X implementation  policy evaluation  Detailed information:  GP conducts the prophylactic check-up at the beginning of academic year and provide the *Personal health prevention card*, PHPC (paper copy) for schoolchildren. A copy of the PHPC is provided to the school doctor/nurse who monitors the children in the same school. The prophylactic examination includes medical history and physical objective status, anthropometric measurements, blood pressure measurement, tests for musculosceletal disorders, laboratory tests of blood and urine.  Activities carried out by the general practitioner during the preventive examination in persons >18 years with risk factors for the development of cardiovascular diseases and type 2 diabetes mellitus: informing the patient about the risk factors for the development of disease; training for self-monitoring and control of weight, blood pressure, heart rate, etc.; giving recommendations for healthy lifestyle - nutrition, physical activity, cessation of harmful habits, reduction of nervous-mental tension; if necessary, consultation with a cardiologist or/and endocrinologist. |
| **Lessons learned** | This agreement is a framework for prevention, treatment and monitoring the health insured population in Bulgaria (primary and hospital health care). |
| **Are you aware of data sources aimed at monitoring variables relevant to this policy / programme?**  **If yes, which ones?** | The National Center for Public Health and Analysis (NCPHA) is a structure of the national health care system and carries out activities on health promotion and disease prevention (incl. T2D or HTN) <https://ncpha.government.bg/?lang=en>;  NHIF <https://www.en.nhif.bg/> |

**A.3 Forms for Greece**

**Questionnaire for each policy / programme document separately**

1. On which country do you report? **Greece**

2. What are the main policy / programme documents in which the vision and major plans for screening, prevention, and management of T2D and HTN in your country have been formulated and T2D or HTN have been mentioned explicitly?

Please mention name and publication year of the policy document and, for each document, the main points concerning screening, prevention, and management of T2D and HTN. You should also include policies on children’s growth/health assessment (if any)

*please fill in the form separately for T2D, HTN and Children’s growth/health* monitoring*.*

See next table for each policy document / programme

| **Name of the policy / programme document (year / url / publication)** | **Name:** Guidelines for the management of patients with Diabetes Mellitus      **Year:** 2021  **URL:** <https://drive.google.com/file/d/1L-zjpv1cYIWlItTDvlW_ljZR4q7esZkx/view>  **Publication:** Hellenic Diabetes Association |
| --- | --- |
| **Focus on** | X T2D  HTN  Children’s growth/ health monitoring |
| **This policy/programme is relevant to:** | the 1^st^ stage screening  the 2^nd^ stage screening  Χ the intervention |
| **In action / Planned** | Χ In action  Planned |
| **In action / Planned date** | 2021 |
| **Initiator / Stakeholder** | government  local authority /community  NGO  Χ other, specify: Scientific Association    Detailed Information:  Hellenic Diabetes Association |
| **Level of policy intervention/ implementation** | Χ national  regional  local/community  school    Detailed Information:  Guiding health care professionals (HCPs) to achieve the best possible result/to treat T2D patients |
| **Category** | legislation  protocol  x guideline  subsidy  programme |
| **T2D and HTN focus (more than one answer possible)** | Screening  Prevention  Management  Screening + Prevention  Screening + Management  Screening + Prevention + Management  X **Prevention + Management** |
| **Policy / intervention targets / policy goals / determinants addressed**  **(more than one answer possible)** | Improving screening of T2D or HTN  Improving prevention of T2D or HTN  X **Improving management of T2D** or HTN  Monitoring growth and development of children in schools or communities  Empowerment  X **Improving (health) services**  Influencing policy  Promoting (please specify below)  Reducing (please specify below)    Detailed information:    This document is the product of the Hellenic Diabetes Association and provides guidelines for the diagnosis, the treatment, and the monitoring of diabetes and accompanying comorbidities. The target of this document is the guidance of HCPs in order to apply the best personalized scheme and achieve the best treatment for the patient, based on scientific clinical evidence (“Evidence Based Medicine”). Dietary, physical activity, glycemic and pharmaceutical targets are specified according to the patient’s profile.    As far as Type 1 Diabetes prevention is concerned, risk prediction is not yet clinically important since the means to successfully prevent the disease, on a large scale, are poor and not proven effective so far.  Regarding Type 2 Diabetes prevention, two possible approaches are presented/discussed: for the general population and for the people with high risk for T2D (ex. obese or pre-diabetic patients), but no specific actions are adopted up to date at a national level.  The former requires central planning meaning the involvement of state (rather than the HCPs) and it’s not proven effective. On the contrary, the effectiveness of the intervention for high-risk patients is well documented, especially for people with impaired glucose tolerance (IGT). High risk individuals are identified after diagnostic test (fasting glucose and sugar curve) for diabetes. Dietary guidelines are an indispensable part of T2D prevention and treatment |
| **Measurement / evaluation / monitoring tools / health professionals involved (e.g. diabetologists, nurses, general practitioners)** | In Greece, there is no specific legislation regarding diabetes management. The present document “Guidelines for the management of patients with Diabetes Mellitus” is the main guide for each health professional during their daily clinical practice for the management of patients with diabetes.    The evaluation and the monitoring of the diabetic patient are performed on a personalized basis. The HCPs, according to the medical history of the patient, the progress, the response to the treatment, generally his/her profile, can prescribe diagnostic tests and medicines and schedule future meetings, based on these national guidelines. |
| **Target group (i.e. general population, children <18,)** | General population |
| **Description** | The document is referred to children, teenagers, and adults with pre-diabetes, T2D, or T1D.  Content: definition, classification, and treatment of diabetes, prevention, dietary and physical activity guidelines, treatment procedures either in a hospital or at home, treatment of diabetes and its comorbidities and implications. |
| **Implementation details**  **(more than one answer possible)** | Phases:  X **problem definition**  agenda setting  policy development  X **implementation**  policy evaluation    Detailed information:  These guidelines provide algorithms for diagnosis, treatment and monitoring of patient’s progress. These algorithms consider the patient’s profile and result in the most appropriate and personalized scheme. |
| **Lessons learned** | As already mentioned, this document constitutes the key guidance for HCPs for diabetic mellitus patients’ management by providing scientific clinical evidence (“Evidence Based Medicine”). |
| **Are you aware of data sources aimed at monitoring variables relevant to this policy / programme?**    **If yes, which ones?** | No, there is no legislation or monitoring of these guidelines on a national level. However through electronic prescription (on the electronic national health care system) the HCP can monitor the prescribed tests and medicines, generally pharmaceutical history of the patient in order to avoid over-prescription and abuse of health services and medicines. |

| **Name of the policy / programme document (year / url / publication)** | **Name:** “Individual student health record (ISHR)”  **Year:** 2014  **URL:**  <https://www.moh.gov.gr/articles/health/dieythynsh-prwtobathmias-frontidas-ygeias/draseis-kai-programmata-agwghs-ygeias/oikogeneiakos-programmatismos/2463-atomiko-deltio-ygeias-mathhth>    **Publication:** |
| --- | --- |
| **Focus on** | T2D  HTN  Children’s growth/ health monitoring |
| This policy/programme is relevant to: | the 1^st^ stage screening  the 2^nd^ stage screening  the intervention |
| **In action / Planned** | In action  Planned |
| **In action / Planned date** | 2014 |
| **Initiator / Stakeholder** | government  local authority /community  NGO  other, specify: Scientific Association    Detailed Information:  Legislation from the government (MoH) |
| **Level of policy intervention/ implementation** | national  regional  local/community  school    Detailed Information: A useful tool for screening and monitoring of student’s health throughout school but with mandatory completion during the registration of children at school at kindergarten, 1^st^ and 4^th^ class of primary school, 1^st^ and 4^th^ class of secondary school. |
| **Category** | legislation  protocol  guideline  subsidy  programme  Can’t tell |
| **T2D and HTN focus (more than one answer possible)** | Screening  Prevention  Management  Screening + Prevention  Screening + Management  Screening + Prevention + Management  Prevention + Management |
| **Policy / intervention targets / policy goals / determinants addressed**  **(more than one answer possible)** | Improving screening of T2D or HTN  Improving prevention of T2D or HTN  Improving management of T2D or HTN  Monitoring growth and development of children in schools or communities  Empowerment  Improving (health) services  Influencing policy  Promoting (please specify below)  Reducing (please specify below)    Detailed information: The ISHR serves the prevention and protection of student’s health. It is also a communication channel between the school units and the doctor. This procedure facilitates the decision making of the teachers for the optimal conduct of classes. |
| **Measurement / evaluation / monitoring tools / health professionals involved (e.g. diabetologists, nurses, general practitioners)** | The ISHR assesses the child’s medical history (including and family history) and vaccination records. What is more, it consists of body measurements and, clinical examination of skin, vision, oral cavity, teeth, heart listening (murmurs, tones, rhythm), respiratory system, abdomen, liver/spleen, genitals, nervous and musculoskeletal system, and scoliosis. The medical examination is performed mainly by paediatrician. If the latter is not available, general doctors or physicians can also carry out the examination. |
| **Target group (i.e. general population, children <18)** | Children at kindergarten, at 1^st^ and 4^th^ class of primary school, and 1^st^ and 4^th^ class of secondary school. |
| **Description** | The role of this document is the screening for the early detection of diseases, and generally the physical and psychosocial support of the child. Further medical control follows in case of special medical indications. It is a prerequisite for the participation of students in sports and other activities at school. |
| **Implementation details**  **(more than one answer possible)** | Phases:  problem definition  agenda setting  policy development  implementation  policy evaluation    Detailed information:  The ISHR is established as a necessary document for the enrolment in the kindergarten, the 1^st^ and 4^th^ class of the primary school, 1^st^ and 4^th^ class of secondary. It also serves as a medical document for the monitoring of student’s health during its presence in primary and secondary education. The document is archived at school’s files and a copy is saved at child’s health booklet. |
| **Lessons learned** | Useful record for monitoring schoolchildren’s growth and development.  Should be electronically kept (currently it is only filled in a paper document) for ease of reference and to serve as a surveillance system. |
| **Are you aware of data sources aimed at monitoring variables relevant to this policy / programme?**    **If yes, which ones?** | According to the legislation, in case student’s health status changes throughout the years, the document is updated under the responsibility of parents or guardians. Since it is a prerequisite for child’s enrollment, it is guaranteed that it will be filled. However, in practice there is not an official monitoring body to assess if this is performed. |

| **Name of the policy / programme document (year / url / publication)** | **Name:** National Action Plan for the Prevention and Treatment of Diabetes Mellitus and its complications      **Year:** 2012  **URL:** <https://docplayer.gr/1875516-Ethniko-shedio-drasis-gia-tin-prolipsi-kai-antimetopisi-toy-sakharodoys-diaviti-kai-ton-epiplokon-toy.html>    **Publication:** |
| --- | --- |
| **Focus on** | T2D  HTN  Children’s growth/ health monitoring |
| This policy/programme is relevant to: | the 1^st^ stage screening  the 2^nd^ stage screening  the intervention |
| **In action / Planned** | In action  Planned |
| **In action / Planned date** | 2012 |
| **Initiator / Stakeholder** | government  local authority /community: Hellenic Diabetic Centre  NGO  other, specify: Hellenic Diabetic Association    Detailed Information:  This document is part of the international campaign “Education and prevention of Diabetes” whose purpose is to inform and educate the public about diabetes. It is created by the Hellenic Association of Diabetes and the National Centre of Research and Treatment of Diabetes in collaboration with World Health Organisation (WHO) and the International Diabetes Federation (IDF). |
| **Level of policy intervention/ implementation** | national  regional  local/community  school |
| **Category** | legislation  protocol  guideline  subsidy  programme  Can’t tell    Detailed information:  It is a national action plan that contains recommendations and policy initiatives but due to various reasons, including political, it is not fully implemented yet. |
| **T2D (more than one answer possible)** | Screening  Prevention  Management  Screening + Prevention  Screening + Management  Screening + Prevention + Management  Prevention + Management |
| **Policy / intervention targets / policy goals / determinants addressed**  **(more than one answer possible)** | Improving screening of T2D or HTN  Improving prevention of T2D  Improving management of T2D  Monitoring growth and development of children in schools or communities  Empowerment  Improving (health) services  Influencing policy  Promoting (please specify below)  Reducing (please specify below)    Detailed information:    Diabetes is a disease that concerns the whole society and requires collective approach. The central goal of the document is to raise awareness not only to the patient and the health care professionals (HCPs), but also to the general public.  To prevent type 2 diabetes, goals for prevention and management are set. More specifically goals for body weight, physical activity, nutrition, smoking, easy access to health units, enhancement of prevention interventions as well as raising awareness for patients and HCPs, providing specialized medical treatment, and upgrading of facilities and equipment. |
| **Measurement / evaluation / monitoring tools / health professionals involved (e.g. diabetologists, nurses, general practitioners)** | The implementation of the goals is evaluated and monitored by the General Secretary of Public Health. |
| **Target group (i.e. general population, children <18,…)** | General population |
| **Description** | The content of the document is:  - definition of diabetes  - risk factors for DM  - epidemiological data  - policy planning for prevention and treatment  - financial burden of DM  - implementation process-funding |
| **Implementation details**  **(more than one answer possible)** | Phases:  problem definition  agenda setting  policy development  implementation  policy evaluation    Detailed information:    The implementation process discussed in the document includes target setting for monitoring the effectiveness of the plan, but not specific steps on how to achieve these targets. The bodies/organisation/institutes involved in programmes for the prevention and management of diabetes will have to report their actions and progress at the Ministry of Health. |
| **Lessons learned** | Not yet implemented, therefore this section cannot be answered yet. |
| **Are you aware of data sources aimed at monitoring variables relevant to this policy / programme?**  **If yes, which ones?** | As already mentioned, the General Secretariat of Ministry of Health will constantly monitor and assess the action plan to ensure efficiency and effectiveness. However, this action plan is not actually implemented. |

| **Name of the policy / programme document (year / url / publication)** | **Name:** European Society of Cardiology (ESC)-European Society of Hypertension (ESH) 2018 guidelines for the management of hypertension (supported by additional explicit guidelines, of 2021, on how to measure the blood pressure(BP)) – translated guidelines by the Hellenic Society of Hypertension      **Year: 2018**  **URL:** <https://hypertasi.gr/UsersFiles/Documents/eshguidelines2018ai.pdf>  (<https://hypertasi.gr/UsersFiles/Documents/eshguidelines2021.pdf>)  **Publication:** Hellenic Society of Hypertension |
| --- | --- |
| **Focus on** | T2D  HTN  Children’s growth/ health monitoring |
| This policy/programme is relevant to: | the 1^st^ stage screening  the 2^nd^ stage screening  the intervention |
| **In action / Planned** | In action  Planned |
| **In action / Planned date** | 2018  (2021) |
| **Initiator / Stakeholder** | government  local authority /community  NGO  other, specify:    Detailed Information:  translated guidelines of the European Society of Cardiology & European Society of Hypertension by the Hellenic Society of Hypertension (HSH) |
| **Level of policy intervention/ implementation** | national  regional  local/community  school    Detailed Information: The European guidelines are translated by the HSH and are used as a milestone, on a national level, for the clinical practice. |
| **Category** | legislation  protocol  guideline  subsidy  programme  Can’t tell |
| **T2D and HTN focus (more than one answer possible)** | Screening  Prevention  Management  Screening + Prevention  Screening + Management  Screening + Prevention + Management  Prevention + Management |
| **Policy / intervention targets / policy goals / determinants addressed**  **(more than one answer possible)** | Improving screening of HTN  Improving prevention of T2D or HTN  Improving management of HTN  Monitoring growth and development of children in schools or communities  Empowerment  Improving (health) services  Influencing policy  Promoting (please specify below)  Reducing (please specify below)    Detailed information:  The current document shares information addressed mostly to health care professionals (HCPs) to update their knowledge regarding the treatment of hypertensive patients. Therefore, goals are settled on the management level. |
| **Measurement / evaluation / monitoring tools / health professionals involved (e.g. diabetologists, nurses, general practitiones)** | The document is addressed to HCPs for the treatment of patients with hypertension. It is explicit on how to measure the BP at several settings and diagnose the patients. An extra updated document, from ESH and HSH, gives practical guidelines on how to measure the BP with or without the presence of a doctor (<https://hypertasi.gr/UsersFiles/Documents/eshguidelines2021.pdf>). It encompasses extensive discussion around various ways of treatment on a pharmaceutical and lifestyle level. |
| **Target group (i.e. general population, children <18,…)** | General population |
| **Description** | The document contains Information about hypertension and comorbidities or other health status (eg. hypertension during pregnancy)    - Diagnosis  - Risk assessment  - Measurement and monitoring of blood pressure  - Treatment goals  - Lifestyle goals |
| **Implementation details**  **(more than one answer possible)** | Phases:  problem definition  agenda setting  policy development  implementation  policy evaluation    Detailed information:  Clear goals regarding lifestyle, BP values, pharmaceutical treatment are settled by the expert commission and are applied by the local health care professionals according to the profile of the patient. A personalized treatment scheme is proposed for the optimal response. |
| **Lessons learned** | This document constitutes the key guidance for HCPs for the management of hypertension by providing scientific clinical evidence (“Evidence Based Medicine”). |
| **Are you aware of data sources aimed at monitoring variables relevant to this policy / programme?**  **If yes, which ones?** | No. |

| **Name of the policy / programme document (year / url / publication)** | **Name:** Arterial Hypertension: committee on the monitoring of pharmaceutical expenditure, the completion of diagnostic therapeutic protocols and the creation of patient’s clinical file/database.      **Year:** 2019  **URL:**<https://www.moh.gov.gr/articles/health/domes-kai-draseis-gia-thn-ygeia/kwdikopoihseis/therapeytika-prwtokolla-syntagografhshs/diagnwstika-kai-therapeytika-prwtokolla-syntagografhshs/5421-diagnwstika-kai-therapeytika-prwtokolla-syntagografhshs-arthriakhs-ypertashs>    **Publication:** General Secretariat of the Ministry of Health – Working Group for Cardiovascular Diseases (CVD) |
| --- | --- |
| **Focus on** | T2D  HTN  Children’s growth/ health monitoring |
| This policy/programme is relevant to: | the 1^st^ stage screening  the 2^nd^ stage screening  the intervention |
| **In action / Planned** | In action  Planned |
| **In action / Planned date** | 2019 |
| **Initiator / Stakeholder** | government  local authority /community  NGO  other, specify:    Detailed Information: The Greek government has established the “Working Group for Cardiovascular Diseases”, for which hypertension is a major risk factor. |
| **Level of policy intervention/ implementation** | national  regional  local/community  school    Detailed Information: The ultimate goal is to make these e-health tools a useful tool for applying the rules of good clinical practice to provide effective health care, but also a tool for collecting big data for making health policy decisions. |
| **Category** | legislation  protocol  guideline  subsidy  programme  Can’t tell |
| **T2D and HTN focus (more than one answer possible)** | Screening  Prevention  Management  Screening + Prevention  Screening + Management  Screening + Prevention + Management  Prevention + Management |
| **Policy / intervention targets / policy goals / determinants addressed**  **(more than one answer possible)** | Improving screening of HTN  Improving prevention of T2D or HTN  Improving management of HTN  Monitoring growth and development of children in schools or communities  Empowerment  Improving (health) services  Influencing policy  Promoting (please specify below)  Reducing (please specify below)    Detailed information: This document was urged by the need of faster diagnosis, optimal treatment, effective monitoring and more efficient management of health resources. |
| **Measurement / evaluation / monitoring tools / health professionals involved (e.g. diabetologists, nurses, general practitiones)** | The applicability of the policies is managed by the “Commission for the monitoring of pharmaceutical expenditure” who is responsible for the approval and implementation of diagnostic and therapeutic prescribing protocols in clinical practice, through their integration in the electronic national health system/application. |
| **Target group (i.e. general population, children <18,…)** | General population |
| **Description** | The content of the document is:  - Definition-classification  - Epidemiological data  - Criteria for treatment  - Treatment goals  - Lifestyle changes  - Pharmaceutical treatment |
| **Implementation details**  **(more than one answer possible)** | Phases:  problem definition  agenda setting  policy development  implementation  policy evaluation    Detailed information: The implementation of advanced pharmaceutical protocols is automatically monitored through the electronic base (digital tools), where patients’ histories are found, and the algorithms for the best treatment protocols are integrated. |
| **Lessons learned** | In Greece there is an explicit, well-designed, and well-established electronic health platform, with integrated algorithms and guidelines, for the screening and treatment of hypertensive patients. |
| **Are you aware of data sources aimed at monitoring variables relevant to this policy / programme?**  **If yes, which ones?** | As described above, the implementation of the relevant protocols is automatically monitored through the electronic base (digital tools), where patients’ histories are found, and the algorithms for the best treatment protocols are integrated. |

**A.4 Forms for Spain**

**Questionnaire for each policy / programme document separately**

1. On which country do you report? Spain

2. What are the main policy / programme documents in which the vision and major plans for screening, prevention, and management of T2D and HTN in your country have been formulated and T2D or HTN have been mentioned explicitly?

Please mention name and publication year of the policy document and, for each document, the main points concerning screening, prevention, and management of T2D and HTN. You should also include policies on children’s growth/health assessment.

*please fill in the form separately for T2D, HTN and Children’s growth/health monitoring.*

See next table for each policy document / programme

| **Name of the policy / programme document (year / url / publication)** | **Name:** Diabetes Strategy of the National System of health  **Year:** 2012  URL: <https://www.aragon.es/documents/20127/674325/Estrategia_en_diabetes_del_SNS_2012.pdf/1e06623b-71b3-48ac-b2c4-ec08184cb062>  Publication: digital |
| --- | --- |
| **Focus on** | X T2D  ☐ HTN  ☐ Children’s growth/ health monitoring |
| This policy/programme is relevant to: | ☐ the 1^st^ stage screening  ☐ the 2^nd^ stage screening  X the intervention |
| **In action / Planned** | X In action  Planned |
| **In action / Planned date** |  |
| **Initiator / Stakeholder** | X government  ☐ local authority /community  ☐ NGO  ☐ other, specify:    Detailed Information: |
| **Level of policy intervention/ implementation** | X national  ☐ regional  ☐ local/community  ☐ school    Detailed Information: |
| **Category** | ☐ legislation  X protocol  X guideline  ☐ subsidy  X programme  ☐ Can’t tell |
| T2D and HTN focus (more than one answer possible) | ☐ Screening  ☐ Prevention  ☐ Management  ☐ Screening + Prevention  ☐ Screening + Management  X Screening + Prevention + Management  ☐ Prevention + Management |
| Policy / intervention targets / policy goals / determinants addressed  (more than one answer possible) | ☐ Improving screening of T2D or HTN  X Improving prevention of T2D or HTN  X Improving management of T2D or HTN  ☐ Monitoring growth and development of children in schools or communities  ☐ Empowerment  X Improving (health) services  ☐ Influencing policy  ☐ Promoting (please specify below)  ☐ Reducing (please specify below)    Detailed information: |
| **Measurement / evaluation / monitoring tools / health professionals involved (e.g. diabetologists, nurses, general practitiones)** | This Strategy involves the Health National System and all the health workers. |
| **Target group (i.e. general population, children <18,…)** | First, patients with diabetes and patients at risk of diabetes. Also, general population for screening. |
| **Description** | The general objective is to contribute to reduce the growing incidence of  Diabetes in Spain, improve life-expectancy and quality of life of patients as well as reducing mortality from Diabetes. It is structured in three blocks of contents: I) Situation Analysis, which addresses the prevalence data, health determinants, complications, mortality, and costs. II) The development of the Strategic Lines: 1) Promotion of healthy lifestyles and primary prevention, 2) Early Diagnosis, 3) Integrated care, 4) Addressing Complications, 5) Diabetes and Pregnancy, 6) Training, research, and innovation. And III) Evaluation. |
| **Implementation details**  **(more than one answer possible)** | Phases:  ☐ problem definition  ☐ agenda setting  ☐ policy development  X implementation  ☐ policy evaluation  Detailed information: |
| **Lessons learned** |  |
| **Are you aware of data sources aimed at monitoring variables relevant to this policy / programme?**  **If yes, which ones?** | No |

| **Name of the policy / programme document (year / url / publication)** | **Name:** Comprehensive Diabetes Mellitus Care Program in the Region of Aragon  Year: 2021  URL: [https://www.aragon.es/documents/20127/47430881/Plan+atenci%C3%B3n+integral+diabetes+mellitus+aragon+2021.pdf/98118fb1-072b-ccf1-0b58-fa61b738208e?t=1621334402172](https://www.aragon.es/documents/20127/47430881/Plan+atenci%25C3%25B3n+integral+diabetes+mellitus+aragon+2021.pdf/98118fb1-072b-ccf1-0b58-fa61b738208e?t=1621334402172)  Publication: digital |
| --- | --- |
| **Focus on** | X T2D  ☐ HTN  ☐ Children’s growth/ health monitoring |
| This policy/programme is relevant to: | ☐ the 1^st^ stage screening  ☐ the 2^nd^ stage screening  X the intervention |
| **In action / Planned** | X In action  ☐ Planned |
| **In action / Planned date** |  |
| **Initiator / Stakeholder** | ☐ government  X local authority /community: Region of Aragon  ☐ NGO  ☐ other, specify:  Detailed Information: |
| **Level of policy intervention/ implementation** | ☐ national  X regional  ☐ local/community  ☐ school    Detailed Information: |
| **Category** | ☐ legislation  ☐ protocol  ☐ guideline  ☐ subsidy  X programme  ☐ Can’t tell |
| **T2D and HTN focus (more than one answer possible)** | ☐ Screening  ☐ Prevention  ☐ Management  ☐ Screening + Prevention  ☐ Screening + Management  X Screening + Prevention + Management  ☐ Prevention + Management |
| **Policy / intervention targets / policy goals / determinants addressed**  **(more than one answer possible)** | ☐ Improving screening of T2D or HTN  ☐ Improving prevention of T2D or HTN  X Improving management of T2D or HTN  ☐ Monitoring growth and development of children in schools or communities  ☐ Empowerment  ☐ Improving (health) services  ☐ Influencing policy  ☐ Promoting (please specify below)  ☐ Reducing (please specify below)    Detailed information: |
| **Measurement / evaluation / monitoring tools / health professionals involved (e.g. diabetologists, nurses, general practitiones)** | A technical group on diabetes was set up at the regional level, made up of endocrinologists, family doctors, nursing, ophthalmologist, vascular surgeons and other professionals involved, whose function is to update and monitor the program. |
| **Target group (i.e. general population, children <18,…)** | General population with Diabetes type 2. All population. |
| **Description** | Comprehensive Diabetes Mellitus Care Program In the Autonomous Community of Aragon with the general objective of improving care throughout the Diabetes Mellitus care process in the Aragon Health System, in order to reduce its incidence and morbidity and mortality and to increase the survival and quality of life of the affected people.  Specific objectives:    - Coordinated attention at the diabetic patients of all the health workers.  - Preventive initiatives, to prevent risk factors aiming the promotion of the healthy lifestyles.  - Active screening.  - To spread to all health areas therapeutic educative programmes for diabetes.  - To collaborate with the patients so they can learn how to manage their diseases  - To include Patient Reported Experience, (Prems) and PROMS (Patient-Reported Outcome Measure, Proms)  - To develop a better approach for the detection and management in the diabetic patients.  - Need of collaboration with the biochemical labs to homogenize the analytic profiles in all the health sectors. |
| **Implementation details**  **(more than one answer possible)** | Phases:  ☐ problem definition  ☐ agenda setting  ☐ policy development  X implementation  ☐ policy evaluation  Detailed information: |
| **Lessons learned** |  |
| **Are you aware of data sources aimed at monitoring variables relevant to this policy / programme?**  **If yes, which ones?** | No |

| **Name of the policy / programme document (year / url / publication)** | **Name:** Nursing care plan In the patient with diabetes mellitus in the Region of Aragon  Year: 2018  URL:https://www.aragon.es/documents/20127/47430881/Plan_enfe_Diabetes.pdf/df64171f-f83e-7d92-1815-4b37b6602807?t=1615371961077  Publication: digital |
| --- | --- |
| **Focus on** | X T2D  ☐ HTN  ☐ Children’s growth/ health monitoring |
| This policy/programme is relevant to: | ☐ the 1^st^ stage screening  ☐ the 2^nd^ stage screening  X the intervention |
| **In action / Planned** | X In action  ☐ Planned |
| **In action / Planned date** |  |
| **Initiator / Stakeholder** | ☐government  X local authority /community: Region of Aragon  ☐ NGO  ☐ other, specify:    Detailed Information: |
| **Level of policy intervention/ implementation** | ☐ national  X regional  ☐ local/community  ☐ school    Detailed Information: |
| **Category** | ☐ legislation  X protocol  ☐ guideline  ☐ subsidy  X programme  ☐ Can’t tell |
| **T2D and HTN focus (more than one answer possible)** | ☐ Screening  ☐ Prevention  ☐ Management  ☐ Screening + Prevention  ☐ Screening + Management  ☐ Screening + Prevention + Management  X Prevention + Management |
| **Policy / intervention targets / policy goals / determinants addressed**  **(more than one answer possible)** | ☐ Improving screening of T2D or HTN  ☐ Improving prevention of T2D or HTN  X Improving management of T2D or HTN  ☐ Monitoring growth and development of children in schools or communities  ☐ Empowerment  X Improving (health) services  ☐ Influencing policy  ☐ Promoting (please specify below)  ☐ Reducing (please specify below)    Detailed information: |
| **Measurement / evaluation / monitoring tools / health professionals involved (e.g. diabetologists, nurses, general practitiones)** | Multidisciplinary team. |
| **Target group (i.e. general population, children <18,…)** | Patients with T2D |
| **Description** | The nurses care plan has two parts:    - The previous evaluation of the health care. This evaluation is a systematized system for gathering relevant information from the patients. In this sense, several tools could be used, like scales, tests, or questionnaires. It is important to highlight that the health care is focusing on the patients and their needs.    - A standardized health plan is the systematized actions of the nurses according to the needs of a specific group of patients based on the results from the previous evaluations. |
| **Implementation details**  **(more than one answer possible)** | Phases:  ☐ problem definition  ☐ agenda setting  ☐ policy development  X implementation  ☐ policy evaluation  Detailed information: |
| **Lessons learned** | A standardized care plan is the summarize of nursing actions according to the care needs presented by groups of patients with the same care area. The collection of information from each patient in the nursing assessment is of great importance in order to know the response of individuals and families to vital processes or health problems, real or potential, that is, to reach the nursing diagnosis. |
| **Are you aware of data sources aimed at monitoring variables relevant to this policy / programme?**  **If yes, which ones?** |  |

**Thank you very much for your cooperation.**
